# PHDD: Corpus of Physical Health Data Disclosure on Twitter During COVID-19 Pandemic

**DOI:** 10.1007/s42979-022-01097-x

**Published:** 2022-04-06

**Authors:** Rana Saniei, Víctor Rodríguez Doncel

**Affiliations:** Ontology Engineering Group, Universidad Politécnica de Madrid, Madrid, Spain

**Keywords:** Corpus, NLP, Personal data detection, Health-related data, General data protection regulation

## Abstract

Health-related information is considered as ‘highly sensitive’ by the European General Data Protection Regulations (GDPR) and determining whether a text document contains health-related information or not is of interest for both individuals and companies in a number of different scenarios. Although some efforts have been made to detect different categories of personal data in texts, including health information, the classification task by machines is still challenging. In this work, we aim to contribute to solving this challenge by building a corpus of tweets being shared in the current COVID-19 pandemic context. The corpus is called *PHDD*(*Corpus of Physical Health Data Disclosure on Twitter During COVID-19 Pandemic*) and contains 1,494 tweets which have been manually tagged by three taggers in three dimensions: *health-sensitivity status*, *categories of health information*, and *subject of health history*. Furthermore, a lightweight ontology called *PTHI(Privacy Tags for Health Information)*, which reuses two other vocabularies, namely *hl7* and *dpv*, is built to represent the corpus in a machine-readable format. The corpus is publicly available and can be used by NLP experts for implementation of techniques to detect sensitive health information in textual documents.

## Introduction

People frequently share their personal information on social media networks without considering the potential consequences and threats to their privacy. Simultaneously, the amount of textual data, of which many are *sensitive* or *personal*, collected by various organizations is overgrowing [7]. Whenever sensitive or personal data is involved, privacy concerns exist as well: in case of online personal data disclosure, users are exposed to a number of threats, such as identity theft, cyber fraud [9], harassment, bullying [16] discrimination in job, credit and visa applications,^1^ and maybe to other unknown long term consequences. When sensitive categories of personal data, such as health-related information are disclosed, the results can be even more serious: insurance companies may increase costs by finding users’ individual and family health history [3], for example. Other results such as stigma, discrimination, and prejudice are also expected, especially when highly sensitive diseases such as mental illnesses, sexually transmitted diseases, or physical disabilities are involved [1, 15].

Personal data resources collected by data controllers are also at risk. This is because of two main reasons: they could be copied and disseminated by unauthorized parties, or they could be published by companies for different purposes such as research or advertisement, without conducting any *sanitization* or *de-identification* process. In these situations, data controllers are not protecting personal data, thus failing to comply with regulations such as the European General Data Protection Regulation (GDPR). In that case, the cost of violation is high: up to 20 million euros or 4% of the total international turnover, whichever is higher, would be considered as a penalty. Consequently, it is of paramount importance for companies to de-identify documents before storing, sharing, or disclosing information; the task in which the first step is to *detect* sensitive and personal data.

Automatic detection of personal data in text documents is a substantial task of interest for both data subjects and data controllers. However, it is one of the important challenges that has not been fully overcome yet. This research work offers a resource that may help solving this challenge: a manually tagged corpus of *health-related information*, which is one of the special categories of personal data mentioned in Article 9 of the GDPR. Thus, the main goal of this research is to collect and categorize tweets that contain *physical health-related information* published during the COVID-19 pandemic. Collected tweets are then tagged in three different dimensions: *physical health sensitivity status*, *categories of health information*, and *information subject of the health history*.

The final corpus is available online^2^ under the FAIR principles [24], and can be used by Natural Language Processing (NLP) experts as a training dataset for the implementation of supervised techniques to detect health-sensitive information in text documents.

We have focused on the COVID-19 domain mainly because of a key reason: it appears that during a pandemic, people consider social media platforms as a way to stay close to each other. They also tend to share their personal and sensitive information to feel more socially connected [2]; the fact that leads to disclosure of types of data necessary for this work. In the same vein, during the COVID-19 pandemic, disclosing health-related information has become increasingly common among users. Many users, for instance, started to write or talk about their own or their family members’ positive test results (*I’m 19 years old and I tested positive for COVID-19*), symptoms (*Before I tested positive for COVID-19, I completely lost my sense of taste. Even black coffee was bland and I couldn’t smell a thing. No congestion either.*), and diseases (*I have lung damage (history of bi-lat PE and subsequent CTED, asthma) and other med issues. If I get COVID-19, I’m likely going to die.*).

This work has been particularly made in the context of a European project called, PROTECT^3^ which investigates methods to protect rights and interests of individuals impacted by the continuous large-scale analysis of personal data. In this framework, an architecture is proposed based on Solid (Social Linked Data)[18], Open Digital Rights Language (ODRL)^4^ and DPV, to assist data subjects handle access to their personal data in a fine-grained manner. For example, considering a use-case in health domain, a user can specify the following privacy preference for Twitter: "I do not want the *medical information* about my *family members*, revealed by me in my tweets, being shared to any other data processors/joint controllers for any purpose". One can simply see that a preliminary step for the data controller to comply with this user-defined policy is to find all the mentioned data in the user-generated texts, in this case, tweets.

Comparing with the previous works, we believe that our work brings two main contributions:

1. Most of the publicly available corpora of health-related information contain medical and clinical records. These documents are usually written by medical staff such as doctors and nurses, who usually utilize a formal language to report patient’s conditions. However, the collected texts in this work, tweets, have informal and diverse language styles, as they are written by people with different educational backgrounds and literacy levels. In addition, other research works that created corpora of health-related information on OSNs, have not published their final corpus. To our knowledge, this is the first effort for building a publicly available corpus of disclosed health information on OSNs during a pandemic, in which text documents are manually tagged from the *privacy* perspective.
2. Other works which have focused on detecting health information disclosure on OSNs mainly studied *self-disclosure* of personal data, in which the author of a piece of text reveals personal information just about herself. In this work, however, we collected tweets revealing health information about any other individual (including family members), taking into account the *identifiability* of the information subject. This is essential as Article 4 of the GDPR defines personal data as any information relating to an identified or identifiable natural person (’data subject’). According to this definition, any health information given about a data subject is tagged in this work as *health-sensitive*.

The rest of this paper is organized as follows. The next section reviews the related works trying to capture sensitive information in online social media networks or other forms of textual documents. The subsequent section explains the methodology for collecting and tagging the tweets. The penultimate section describes the resulted corpus, and finally, some future works are described and the paper is concluded.

## Related Work

There have been several works which created corpus of text documents containing different types of personal data. Tesfay et al. [21], for example, created a dataset of 319,210 tweets containing different categories of sensitive personal data. Forty-two participants manually annotated tweets in two levels: first, sensitivity of the tweets, and second, the category of personal information in the tweets tagged as sensitive. To define the categories of personal information, they utilized the existing works such as [4, 23], and the list of sensitive categories of data outlined in Article 9 of the GDPR. In another work, Mao et al. [14] studied three types of sensitive information leakage on Twitter: vacation tweets (revealing travel plans and specifically, the time people are away from home), drunk driven tweets (containing sensitive information published by drunk users), and diseases tweets (containing information about oneself/others diseases). For the latter category, a list of diseases was prepared using the disorders outlined in the Mendelian Inheritance in Man (OMIM) catalogue,^5^ along with the title of 8 other common diseases (tumors, depression, cancer, obesity, HIV, HPV, AIDS, and diabetes), which together led to using 390 types of diseases as keywords for filtering the tweets. In a more recent work, Keküllüoglu et al. [12], studied the leakage of private life events (e.g., having a baby, starting a relationship, marriage, LGBTQ-related, and surgery) in Twitter using the phrase *happy for you* as the seed for finding relevant tweets. In another work, Ghazinour et al. [6] presented a tool for detection of health-related personal information in the online social network, *MySpace*. For this goal, they generated a dataset of posts following these two steps: first, they found posts including personal health information using the terms in two medical terminologies; *MedDRA* and *SNOMED*. Then, annotators reviewed about 12,000 posts and labeled founded medical terms in three main categories: *PHI* (terms disclosing personal health information), *HI* (terms related to health information but not necessarily personal ones), and *NHI* (terms with no health or medical meaning). While they considered health information about *identified* people as PHI, for the sake of simplicity they ignored the cases when the information subject is *identifiable*; for example, when the information subject is the author’s family member. In another work, presented by Sokolova et al. [20], a system was developed for the detection of personal health information (PHI) in heterogeneous texts. The main objective in this work was to protect the privacy of data subjects by preventing the leakage of PHI such as prescribed drugs, symptoms, and diseases. For their experiments, they created a corpus of 2,852 files, collected from two p2p file sharing networks.

As mentioned in "Introduction", most of the publicly available corpora of health-related information are of two types: first, datasets containing medical and clinical records which are usually written by medical staff using formal languages to report patient’s conditions (see, for example, MIMIIC-III [10], i2b2 2014,^6^ datasets), and second, datasets containing health news or scientific medical texts. Some examples are an unlabeled corpus of 2434 nursing notes,^7^ MuchMore Springer Bilingual Corpus^8^ which is a labeled corpus of scientific medical abstracts in English and German from the Springer website , Ohsumed collection^9^ which is a labeled corpus of medical abstracts from the Medical Subject Heading (MeSH)^10^ categories, and a dataset of health news on Twitter [11].

There have also been other research works in this area specifically focused on the pandemic. For example, a recent work proposed by Blose et al. [2] analyzed the English-language Tweets between March 1 and April 3, 2020, to capture different types of self-disclosing by users in the context of the COVID-19 pandemic. In this approach, first-person pronouns, subject–verb–object triples, named entity recognition, rule-based matching, and self-disclosure dictionaries were used to detect self-disclosure in texts. They found that approximately 18% of the tweets in their dataset contain self-disclosure elements. Their results also suggested a meaningful relation between users’ online self-disclosure behaviors and situational contexts during a crisis. Other works have been done over the course of COVID-19 that aim to capture and analyze different types of self-disclosing information on Twitter, albeit not for privacy-preserving and personal data protection purposes, instead, to get further insight into the effect of a pandemic on societies, prepare statistics, or explore different aspects of the virus. For example, Guntuku et al. [8] studied Twitter to analyze changes in the used languages by users during the pandemic across different states of the USA, with a special focus on estimating mental health and finding symptoms. Their results showed that overall mental health in the US decreased after the declaration of emergency in the country, compared with the same time in 2019. They could also capture emerging symptoms such as skin lesions, change in smell/taste, and body ache in their dataset.

Detecting self-reported symptoms of COVID-19 on Twitter is the subject of some other research works such as [13, 19]. In [19], the authors, first, analyzed symptoms reported by positive-tested users on Twitter. Then they semi-automatically mapped different expressions of symptoms to standard concept IDs in the Unified Medical Language System, using their meta-lexicon and the National Center for Biomedical Ontology BioPortal^11^ (e.g., ’*pounding in my head’* = Headache). They found 1002 symptoms expressed by 203 users and categorized them into 46 groups. Finally, they compared the distribution of each group of symptoms with those reported in clinical studies. Lastly, Mackey et al. [13] suggested an unsupervised machine-learning method for capturing self-reporting symptoms on Twitter and characterizing experiences associated with testing (e.g., lack of access to testing) and recovery from the disease. The dataset in this work was generated using COVID-19 general filtering keywords along with specific terms related to the their study (e.g., *testing kit*, *diagnosed*, *symptoms*, *isolating*, etc.). Then, three groups of topics have been identified, namely, *symptoms*, *recovery*, and *testing experiences*. Then, the collected tweets in each of these groups have been manually annotated for both first and secondhand reports.

Considering the works mentioned above, we can see that this is the first effort for building a corpus of user-generated, health-related information, which is tagged from the privacy perspective. The most similar work to ours is presented by [6], although their tagging criteria are not comprehensive enough to capture the health-sensitive tweets which contain information about *identifiable* information subjects. On the other hand, other works presented in the context of the COVID-19, such as [19], did not analyze texts from the privacy point of view and just focused on collecting relevant information about the disease.

## Methodology

### Domain Specification

Twitter is a micro-blogging platform for publishing content and communicating spontaneously. We choose Twitter as our data source in this work because of three main reasons: first, unlike the other OSNs, such as Facebook, users are not restricted to using their real identity/name on Twitter until they do not impersonate anyone else. Hence, they can tweet in anonymity, which is an encouraging factor in posting sensitive data, including health information. Second, tweeting is accepted publicly as a quick way to talk about, and trend topics of running events using hashtags. Also not surprisingly, "#COVID19" is at the top of the list of most-used hashtags on Twitter in 2020,^12^ which makes the platform a good option for extracting user-generated data about the disease. Furthermore, the social data published on Twitter is accessible through the Twitter API,^13^ which makes it possible to easily filter and retrieve data. Using this API, we aimed to retrieve tweets that have been published during the pandemic and contain some forms of physical health data disclosure. For this goal, we referred to the definition of the "Protected Health Information" (PHI), provided by the Health Insurance Portability and Accountability Act (HIPAA)^14^: *"individually identifiable health information related to the past, present, or future physical or mental health status or condition of an individual"*. We have considered this definition in articulating our tagging criteria.

Based on HIPAA rules, all the information such as diagnoses, medical test results, treatment, and prescription information are considered sensitive health information. Also, information about family medical history, once it is disclosed, is part of the individuals’ protected health history. Considering this guideline, we decided to mainly focus on three types of health-related information that users tend to share more on Twitter during the pandemic: (i) positive result of their tests (mainly COVID-19 tests); (ii) physical symptoms, and (iii) their health history (diseases and other physical conditions). Interestingly, we found that many users have not only shared their-own health/medical information in these three categories, but also their family members’. For instance, many users have shared their family members’ positive COVID-19 test results and the name of diseases or special health conditions they have or had in the past. The latter became a trend by many users whose family members (or even themselves) belong to the high-risk category to COVID-19.

### Collecting Data

There are several ready-to-use Twitter datasets related to COVID-19. Our primary dataset for collecting data is the public coronavirus Twitter dataset proposed by Chen et al. [5], which is available online.^15^ This resource contains Tweets ID in different languages associated with the new coronavirus. As of February 2021, the resource had more than one milliard tweets from languages such as English, Spanish, Arabic, and German. In this research, we filtered tweets based on their language and just collected content in English.

A python script, Twarc,^16^ was used to retrieve the content of tweets based on their IDs. Then, to collect tweets in each category of physical health information mentioned earlier, we utilized the following methods:

- Test Results We applied the first level of filtering using some keywords such as *test*, *negative*, and *positive* with at least one of the pronouns *I, we, my, and us* for discovering individual test result disclosure. Other pronouns such as *they, he, and she* and different nouns representing one of the family members, namely *mother, father, grandmother, grandfather, sister, brother, uncle, aunt, wife, husband, etc.* have also been used to retrieve information disclosure about family members. The second layer of filtering we applied is using some regular expressions started with one of the pronouns or nouns representing family members, following the patterns *test[ed?|s?] positive* and test.*came back positive. To avoid being too exclusive and maintain other expressions people used to announce they tested positive for COVID-19, we saved the results of the second filtering layer together with a random number of tweets obtained in the first one.
- Symptoms To obtain symptoms related to COVID-19, the regular expression *symptom(s)? (are|is)* and (has|have) symptom(s)? have been used together with the pronouns and nouns mentioned above. However, these regular expressions do not retrieve all the sentences containing users’ reported symptoms. The main challenge here is that people usually use different and sometimes informal language to talk about their symptoms, which are not always predictable. To overcome this challenge, we took advantage of the work presented by [19]. In this work, the tweets containing symptoms are collected by, first, finding users who have reported they tested positive for COVID-19, and second, collecting their reported symptoms during the infection period. We have also used as filtering keywords the common symptoms of the disease, reported in World Health Organizations (WHO)^17^ guidelines.
- Diseases The hashtag *#highRiskCovid19* and its equivalents have been trending on Twitter, highlighting the stories of people with serious diseases or conditions (such as diabetes, heart disease, lung disease, etc.), which place them in a high vulnerability to COVID-19. Accordingly, we retrieved tweets containing these hashtags to retrieve tweets with information about people’s diseases. Names of specific conditions and diseases, reported by *WHO* and other organizations such as *Center for Disease Control and Prevention*,^18^ have also been used as keywords to collect tweets belonged to this category.

After collecting data, we replaced re-tweets with the original tweets.

**Fig. 1:**
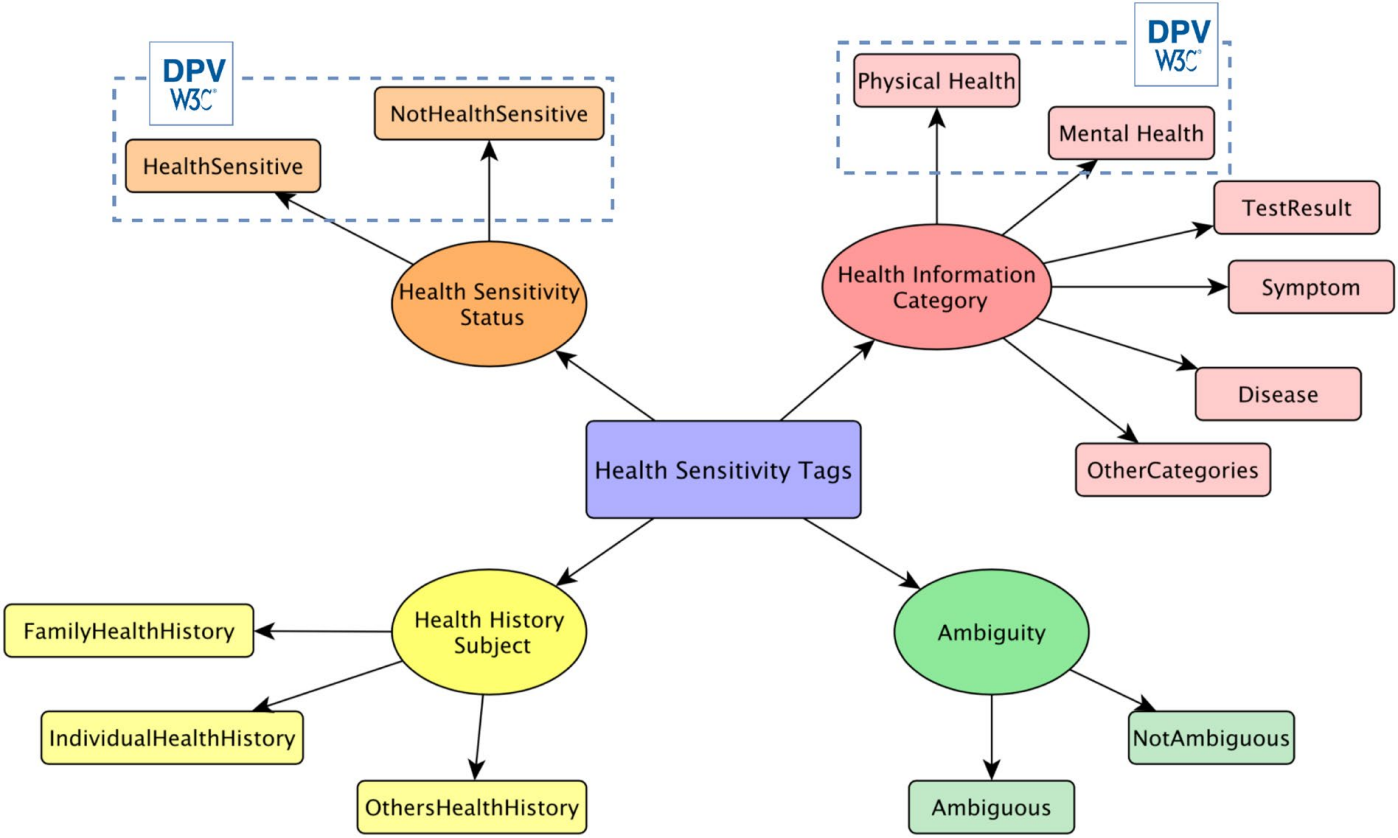
Used labels in the tagging process of the collected tweets. Three labels have been applied to each tweet: 1) Health Sensitivity Status, which shows whether a tweet contains health information or not, 2) Health Information Category, which determines the type of health information provided in a tweet, and 3) Health History Subject, which shows the subject of the health information. If a tweet is unclear or is impossible to infer its sensitivity status, it is tagged as Ambiguous

**Table 1:** Tagging criteria for the tweets, along with some examples for each one

Dimension#1	Health sensitivity status			
	Health sensitive (HS)		Not health sensitive (NHS)	
Criteria	Tweet containing any information about an identifiable individual’s physical health conditions,(e.g., test results, symptoms, diseases, treatments, prescription) currently or in the past		Tweets containing no information about physical health of identifiable individuals	
	Health Sensitive tweets could have information about the writers themselves, their family members, or any other identifiable individual		Tweet containing news or recent findings related to COVID-19	
Example	My mom is immunocompromised from her breast cancer, please wash your hands		I wish/dreamt I tested positive for COVID19	
	My COVID-19 test came back positive yesterday		Canada/1 patient in Toronto preliminary tested positive for coronavirus and has been classified as a presumptive case	
	“I tested positive for Coronavirus”, says Health Minister of Iran		Thanks God! my test came back negative!	
	I’m asthmatic and most of my family is immunocompromised, COVID-19 could kill all of us!		Four children in Los Angeles County diagnosed with a rare inflammatory disease have tested positive for COVID-19 through antibody testing, officials said Monday	

Dimension#2	Health information category			
	Test result (TES)	Symptom (SYM)	Disease (DIS)	Other types (OTH)
Criteria	Health sensitive tweets containing any information regarding positive test results for COVID-19 or any other diseases	Health sensitive tweets containing any specific symptoms	Health sensitive tweets containing names of individuals’ diseases or any physical health conditions (e.g., being immunocompromised), currently or in the past,treated or not	Health sensitive tweets with some information not falling into the mentioned categories. (For example, treatment history, prescription, used medications, etc.)
	Tweets containing only negative test results are tagged as non health-sensitive (N-HS)			
Examples	I’m 22 years old and I tested positive for COVID-19. I’ve been debating on posting, but I want to share my experience especially with those around my age	My dad is experiencing severe fatigue and chronic headaches because of his COVID-19	I’m actually gonna die if i get coronavirus because I have asthma and my lungs are weakened from previously having pneumonia	I spent 8 days in hospital hooked up to oxygen as I could no longer breathe on my own, scared not knowing what was going to happen. Luckily I got through it and I’m starting to feel a lot better now
	My parents and I tested positive for COVID-19. I didn’t have any symptoms at all but still went to get tested. I did nothing else but go to work and come home. Today I have a small headache and that is about it. Were all doing fine and I’m sure we will be fine. Be safe people!	My 9 yr old niece started school in GA on Monday.... Thursday she came home w/a cough and fever which spiked to 104.5$^{\circ }$. We are waiting on her test results and praying it isn’t #COVID19	I’m 46. I have cancer. I might die if I get the Coronavirus and that isn’t hyperbole. And there are more people like me under 50 than most people think. Especially since many cancer patients look “healthy” these days	My uncle has cancer. He waited 3 weeks to find out (for sure): at least a week longer than usual. Then, last week, he was told he’s too frail for major surgery so needs radiation therapy. Today, he was told his first consultation won’t be till the end of JULY. Why? Covid

Dimension#3	Health history subject			
	Individual Health History(IND)	Family health history (FAM)		Others (OTH)
Criteria	Health sensitive tweets containing above-mentioned information about the writer him/herself	Health sensitive tweets containing information about the writers’ family member (explicitly mentioned in the tweets)		Health sensitive tweets containing information about identifiable individuals (for example, by their name or twitter account) who do not have a family relationship to the writer or their family relationship is unknown
Examples	My doctor just told me that I tested positive with Corona	For your information my sister is a chronic smoker, my sisters wife has asthma and my dad has cancer.I lived through covid and one of my sisters and my mom would most likely be fine. But everyone else? Who knows?		Cristiano Ronaldo has tested positive for Coronavirus
	My name is Corinne Kahn. I am #HighRiskCovid19. I received a #kidneytransplant 2 weeks ago			Dilip Kumar’s younger brother, Aslam Khan, passes away at 88 after testing COVID-19 positive

### Tagging the Corpus

We collected 22,331 tweets after applying different levels of filtering explained in "Collecting data", from which we randomly selected and tagged 1494 ones based on the criteria explained in this section.

Every Tweet is tagged in 3 different dimensions represented in Fig. 1. In the *Health Information Category* and *Information Subject* dimensions, more than one tag is possible. If a tweet is unclear or is impossible to infer its sensitivity status, it is tagged as *Ambiguous*. Table 1 gives a brief explanation of the tagging criteria used for tagging the tweets in each of the three mentioned dimensions, as well as a few example for each one.

Four different people intervened in the tagging process to determine if the tweets belong to the categories mentioned above. They acted independently by having just a common tagging criteria document. We asked taggers to check tweets as *ambiguous* if they are not sure about the right tag(s). After finishing the tagging process by taggers 1, 2, and 3, the fourth person reviewed all the tags, with a particular focus on the dissimilar tags for the same tweets and tweets marked as ambiguous: if an agreement is reached on any of these cases, the tags are updated consequently, otherwise, when a consensus is not reached, the tweet is tagged as *ambiguous*. We decided to keep these ambiguous tweets in the final corpus with all the dissimilar tags assigned to them. Below, for example, you can see a tweet which is tagged as not-health sensitive by tagger 1 and health sensitive by taggers 2 and 3:

"*My mom had a rheumatologist appointment this week. Everyone who goes there for an appointment is immunocompromised. But they didn’t take temperatures at the door. And they let patients sit in waiting room w/someone who was coughing violently. In a major metropolitan area.*"

The following tweet, which could have different interpretations, is also tagged as *ambiguous*:

*Q: I’m not an essential worker but I tested positive for COVID-19. What should I do? A: You may qualify for paid leave under PGH or PHL paid sick laws, and/or the Emergency Paid Sick Leave Act*

The reliability of agreements between the taggers for the first dimension (health-sensitivity) is 0.96 which is considered acceptable [22]. This is calculated using the Fleiss kappa measurement implemented in python.

## PHDD Corpus

### Corpus Description

Linguistic information of the final corpus, called *PHDD*(*Corpus of Physical Health Data Disclosure on Twitter During COVID-19 Pandemic*), can be found in Table 2, while Table 3 represents the statistics on each tag in the three discussed dimensions. Based on Twitter’s privacy policy, it is restricted to publish the content of the tweets. Therefore, we only published identifiers of the collected and tagged tweets, as well as the associated source code, in the dedicated GitHub page^19^ of this work.

**Table 2:** Total and average (per tweet) statistics on the corpus

	Total	Average
Tweets	1494	–
Sentences	5126	3.42
Tokens	63,029	42.15
Hashtags	685	0.45
Mentions	855	0.57
URLs	537	0.36
Verbs	7437	4.97
Nouns	11,114	7.43
Proper nouns	3134	2.10
Pronouns	6778	4.53
Adjective	3867	2.58
Adverbs	3143	2.10

POS information was obtained using Stanza [17], while patterns were used to detect URLs (‘www.*’/‘http*’), hashtags (*‘#’*), and mentions (*‘@’*)

**Table 3:** Statistics on the corpus for *Health Sensitivity Status* , *Health Information Category*, and *Health History Subject* dimensions

*Health sensitivity status (%)*		
HS	656	41.12
NHS	797	47.86
Ambiguous	38	1.13
*Health information category (%)*		
TES	202	30.03
SYM	192	47.86
DIS	346	51.30
OTH	51	6.00
*Health history subject (%)*		
IND	351	56.49
FAM	244	38.96
OTH	52	7.14

For each dimension, the percentage and number of tags are represented. Percentages of tags for *Health Information Category*, and *Health History Subject* dimensions are calculated in tweets tagged as *HS*. (*Health Sensitive* (HS), *Not Health Sensitive* (NHS),*Test* (TES), *Symptom* (SYM), *Disease* (DIS), *Other types* (OTH), *Individual health history* (IND), *Family health history* (FAM), *Others/Other Categories* (OTH))

### Publishing the Corpus as Linked Data

In addition to the simple XLSX format, the corpus is also available in RDF format to pursue a richer presentation. To this end, we built a lightweight ontology called *Privacy Tags for Health Information (PTHI)* to represent the aforementioned dimensions of an information object, in this case, a tweet. PTHI also covers two other categories of privacy tags, namely *Sensitivity* and *Confidentiality*. To represent these dimensions, we reused *Sensitivity* and *Confidentiality* ontologies; which are parts of the *Privacy and Security* ontology provided by the *Health Level Seven (HL7)*^20^ International Standards. Sensitivity Ontology specifies different types of sensitivity for a record in health-domain (for example, *HIV* for *HIV/AIDS* information sensitivity), while the Confidentiality ontology represents different levels of confidentiality (e.g., low, medium, normal, restricted, etc.) needed for an information object.

**Fig. 2:**
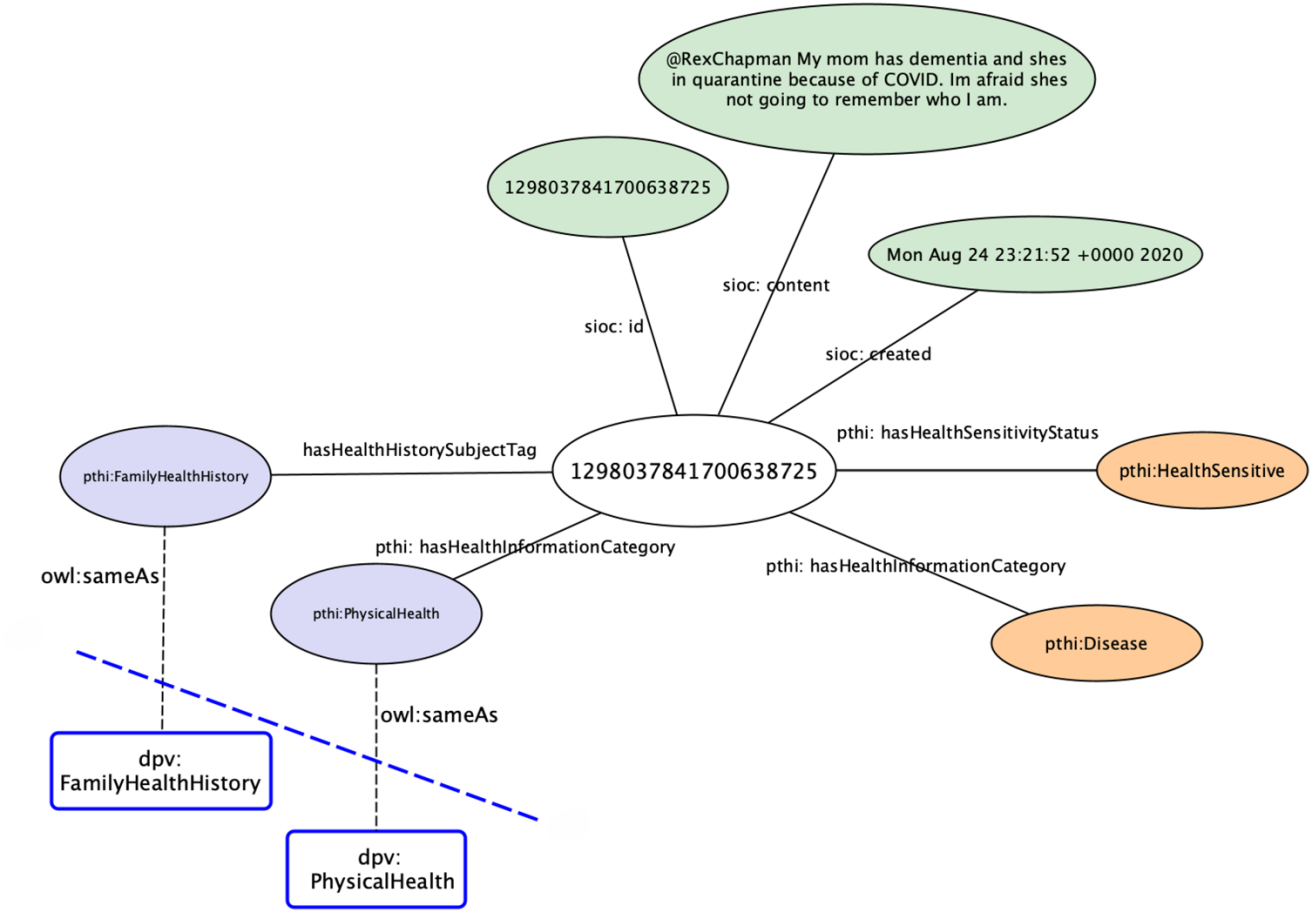
A sample tweet represented by *pthi*, *dpv*, and *sioc*

In the PHDD corpus, we reused PTHI, *DPV*(Data Privacy Vocabulary)^21^ and *SIOC*(Semantically-Interlinked Online Communities)^22^ vocabularies. SIOC provides vocabularies necessary to represent online content and is used to describe Twitter posts, while DPV is a vocabulary providing the essential terms to represent the processing of personal data according to regulations such as GDPR. To represent the tags *IND* and *FAM* the DPV terms *IndividualHealthHistory* and *FamilyHealthHistory* are used. Furthermore, tweets with "HS" tag are represented using the term *PhysicalHealth* in DPV. A sample record in the corpus is represented in Fig. 2. The PTHI ontology^23^ and PHDD corpus^24^ are available online under the FAIR principles to the research community.

## Conclusion and Future Work

In this paper, we presented (1) the PHDD corpus; a corpus of the physical health information disclosed in Twitter in the context of the COVID-19 pandemic, (2) the methodology for collecting, tagging, and building the corpus and (3) the PTHI lightweight ontology for publishing the corpus in the RDF format. The generated corpus will be used in future works to train an NLP tool for detection of health-related information in text documents, supporting the implementation of a fine-grained access control mechanism for Solid (Social Linked Data) [18] using ODRL and DPV. The corpus will also be available in the ELRA Catalogues.^25^

## Data Availability

The data that support the findings of this study are openly available in zenodo at https://doi.org/10.5281/zenodo.4538359.

## Footnotes

1 https://theconversation.com/four-ways-your-google-searches-and-social-media-affect-your-opportunities-in-life-96809.

2 https://protect.oeg.fi.upm.es/def/phdd/.

3 https://protect-network.eu/.

4 https://www.w3.org/TR/odrl-model/.

5 http://www.ncbi.nlm.nih.gov/Omim/omimfaq.html#db_descr.

6 https://www.i2b2.org/NLP/DataSets/.

7 https://physionet.org/.

8 https://muchmore.dfki.de/resources1.htm.

9 https://link.springer.com/article/10.1007/s40815-017-0327-9#Fn12.

10 https://www.nlm.nih.gov/mesh/.

11 https://bioportal.bioontology.org/.

12 https://blog.twitter.com/en_us/topics/insights/2020/spending-2020-together-on-twitter.html.

13 https://developer.twitter.com/en/products/twitter-api.

14 https://www.hhs.gov/hipaa/index.html.

15 https://github.com/echen102/COVID-19-TweetIDs.

16 https://github.com/DocNow/twarc.

17 https://www.who.int/.

18 https://www.cdc.gov/.

19 https://github.com/rsaniei/tweets-privacy-checker.

20 https://www.hl7.org/.

21 https://dpvcg.github.io/dpv/.

22 https://www.w3.org/Submission/sioc-spec/.

23 https://protect.oeg.fi.upm.es/def/pthi/widoco/index-en.html.

24 https://protect.oeg.fi.upm.es/def/phdd/.

25 http://www.elra.info/en/catalogues/.

## Code Availability

The code that supports the findings in this study is openly available in https://github.com/rsaniei/tweets-privacy-checker.
